# Structural Basis for Vascular Endothelial Growth Factor Receptor Activation and Implications for Disease Therapy

**DOI:** 10.3390/biom10121673

**Published:** 2020-12-15

**Authors:** Faheem Shaik, Gary A. Cuthbert, Shervanthi Homer-Vanniasinkam, Stephen P. Muench, Sreenivasan Ponnambalam, Michael A. Harrison

**Affiliations:** 1 School of Molecular and Cellular Biology, University of Leeds, Leeds LS2 9JT, UK; s.ponnambalam@leeds.ac.uk; 2 William Harvey Research Institute, Barts and The London School of Medicine and Dentistry, Queen Mary University of London, London EC1M 6BQ, UK; 3 Faculty of Medicine and Health, University of Leeds, Leeds LS2 9JT, UK; Gary.cuthbert@nhs.net (G.A.C.); shervanthi.homer-vanniasinkam@nhs.net (S.H.-V.); m.a.harrison@leeds.ac.uk (M.A.H.); 4 School of Biomedical Sciences, University of Leeds, Leeds LS2 9JT, UK; s.p.muench@leeds.ac.uk

**Keywords:** angiogenesis, VEGFR, receptor tyrosine kinase, signal transduction, cancer, bevacizumab, aflibercept, sunitinib

## Abstract

Vascular endothelial growth factors (VEGFs) bind to membrane receptors on a wide variety of cells to regulate diverse biological responses. The VEGF-A family member promotes vasculogenesis and angiogenesis, processes which are essential for vascular development and physiology. As angiogenesis can be subverted in many disease states, including tumour development and progression, there is much interest in understanding the mechanistic basis for how VEGF-A regulates cell and tissue function. VEGF-A binds with high affinity to two VEGF receptor tyrosine kinases (VEGFR1, VEGFR2) and with lower affinity to co-receptors called neuropilin-1 and neuropilin-2 (NRP1, NRP2). Here, we use a structural viewpoint to summarise our current knowledge of VEGF-VEGFR activation and signal transduction. As targeting VEGF-VEGFR activation holds much therapeutic promise, we examine the structural basis for anti-angiogenic therapy using small-molecule compounds such as tyrosine kinase inhibitors that block VEGFR activation and downstream signalling. This review provides a rational basis towards reconciling VEGF and VEGFR structure and function in developing new therapeutics for a diverse range of ailments.

## 1. Introduction

Vasculogenesis is the de novo formation of a vascular network whereas angiogenesis is sprouting of new blood vessels from pre-existing ones. Both processes are highly dependent on regulation by vascular endothelial growth factors (VEGFs) and their interaction with membrane receptors expressed on different cell types. One class of receptor–ligand interaction occurs on the endothelium, a specialised cell type that lines all blood vessels in metazoan species from man to fish [1,2]. Physiological angiogenesis is an essential feature of embryogenesis, wound healing and tissue regeneration. However, abnormal angiogenesis is associated with a variety of diseases such as tumour neovascularisation, diabetic retinopathy, rheumatoid arthritis and age-related macular degeneration [3]. Tumour neovascularisation is triggered by cancer cells to stimulate supply of nutrients and enable metastasis. In the absence of a functional blood supply, tumours are either dormant or necrotic [4,5]. Angiogenesis is thus a major contributory factor in the growth and spread of a variety of cancers which cause substantial mortality.

The VEGF family has complexity with multiple isoforms encoded by each VEGF-related gene, and differences in biological activity between closely related variants [6]. These ligands bind to VEGFRs which belong to the type IV receptor tyrosine kinase (RTK) family and comprises of VEGFR1 (Flt1), VEGFR2 (KDR, Flk1) and VEGFR3 (Flt4) [7]. VEGFRs possess a cytoplasmic tyrosine kinase (TK) domain which regulates signal transduction pathways linked to cell proliferation, migration, metabolism, vasodilation, blood vessel formation and remodelling [7,8]. VEGFR1 and VEGFR2 play important roles in physiological and pathological angiogenesis, whereas VEGFR3 is mainly involved in lymphangiogenesis [9,10]. This review focuses on the mechanistic basis for VEGFR activation and function linked to the current portfolio of the drugs that target such molecules.

## 2. VEGF Ligands and Receptor Diversity

VEGF ligands regulate embryogenesis, blood vessel development, sprouting and homeostasis [11]. Seven VEGF-related genes encoding numerous splice isoforms have been identified. In most metazoan species, genes encoding VEGF-A, VEGF-B, VEGF-C, VEGF-D and placental growth factor (PIGF) are present [12,13]. VEGF-E is encoded by a parapoxvirus genome and VEGF-F is secreted in some snake venoms [14,15]. VEGFs also interact with heparan sulphate proteoglycans (HSPs), neuropilin 1 and 2 co-receptors (NRP1 and NRP2). Furthermore, the growth factors are dimeric in vivo (i.e., divalent molecule), sharing the same basic fold, and can form both homo- and hetero-dimers with each other [16,17,18,19].

### 2.1. VEGF-A

**Figure 1:**
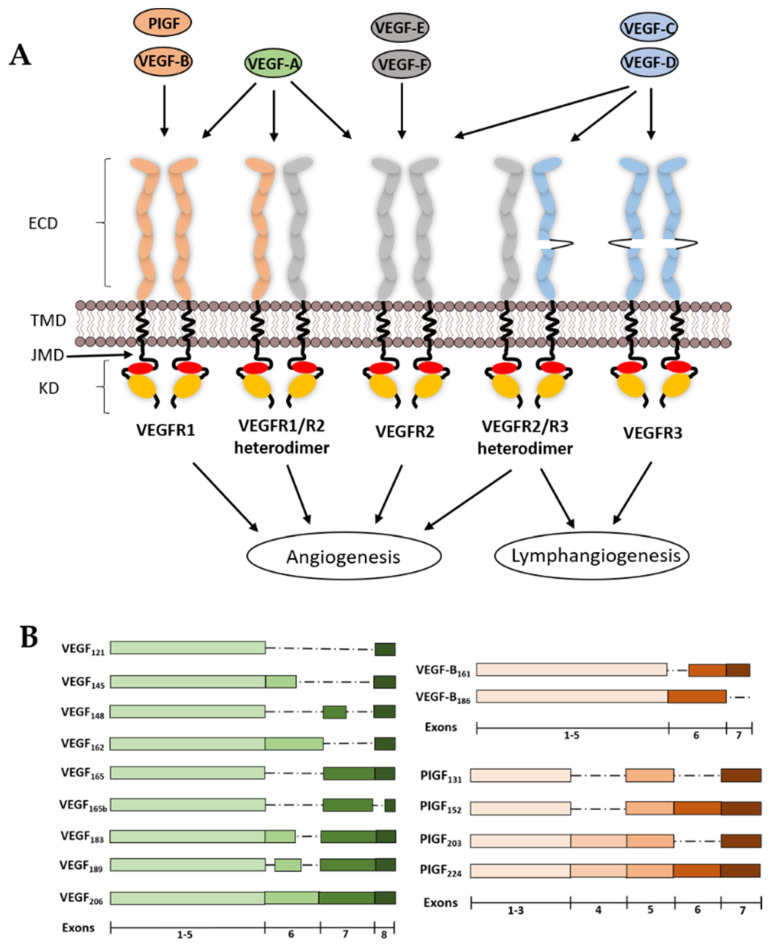
(**A**) Schematic representation of vascular endothelial growth factor receptor (VEGFR) organisation and ligand specificity. All vascular endothelial growth factors (VEGFs) bind to three receptor tyrosine kinases (RTKs): VEGFR1, VEGFR2 and VEGFR3. VEGFRs contain an extracellular domain (ECD), transmembrane domain (TMD) and a cytoplasmic domain which is further divided into juxtamembrane domain (JMD) and kinase domain (KD). VEGFRs form homo- and hetero-dimers upon ligand binding. VEGFR1 and VEGFR2 form heterodimers, whereas VEGFR3 forms a heterodimer with VEGFR2. VEGF-A, placental growth factor (PIGF) and VEGF-B bind to VEGFR1 homodimers, VEGF-A bind to VEGFR1 homo-, VEGFR2 homo- and VEGFR1/R2 hetero-dimers, VEGF-E and VEGF-F recognise VEGFR2 homodimers and VEGF-D and VEGF-D only bind to VEGFR3 homodimers. The fifth extracellular immunoglobulin-like domain of VEGFR3 is replaced with disulphide bonds. VEGFR1 and VEGFR2 play a major role in angiogenesis, whereas VEGFR3 is mainly involved in lymphangiogenesis. (**B**) Isoforms of VEGF ligands: alternative splicing of VEGFA primary RNA transcript can produce at least 9 isoforms if VEGF-A. VEGF-B can exist as two isoforms and PlGF can exist as four isoforms.

VEGF-A was also known as vascular permeability factor (VPF) and is usually a homodimer formed by the arrangement of two anti-parallel VEGF-A monomers with a receptor binding site at each pole or C-termini. VEGF-A can also form heterodimers with VEGF-B and PIGF [20] and specifically binds to VEGFR1 and VEGFR2 expressed on endothelial cells, and can also bind the NRP1 and NRP2 co-receptors expressed on the vascular endothelium and neurons [21] (Figure 1A). The pattern of VEGF-A expression is dependent on the VEGF-A gene which encodes at least 8 exons. Alternative splicing of the primary RNA transcript can generate at least 9 VEGF-A splice isoforms such as VEGF-A_121_, VEGF-A_145_, VEGF-A_148_, VEGF-A_162_, VEGF-A_165_, VEGF-A_165b_, VEGF-A_183_, VEGF-A_189_ and VEGF-A_206_ (Figure 1B). The expression of VEGF-A is known to be upregulated by hypoxia, *p53* allele polymorphisms, thyroid stimulating hormone (TSH) and nitric oxide (NO). The VEGFA promoter is dependent on activation by the hypoxia inducible factor-1 (HIF-1), which is composed of α and β subunits [22,23]. All the VEGF-A splice isoforms stimulate VEGFR tyrosine kinase activity except for VEGF-A_165b_, which has been proposed to negatively regulate VEGFR activity [23]. VEGF-A is the most potent pro-angiogenic growth factor compared to other VEGFs and the deletion of the VEGFA gene in mice shows embryonic lethality even with the loss of only a single allele [24].

### 2.2. VEGF-B 

VEGF-B is encoded by the VEGFB locus and consists of eight exons and six introns. Alternative splicing of the VEGF-B primary RNA transcript generates two splice isoforms, VEGF-B_167_ and VEGF-B_186_ (Figure 1B). These VEGF-B isoforms only bind to VEGFR1, but not VEGFR2 or VEGFR3. Upon VEGF-B binding and activation of VEGFR1, it induces poor signalling and is found to have negligible effect in inducing blood vessel growth [13,25]. The role of VEGF-B is still unclear, but mice lacking a functional VEGFB locus have smaller hearts, impaired angiogenic response and decreased capillary density [26]. However, VEGF-B is essential for blood vessel survival [27].

### 2.3. VEGF-C and VEGF-D

There are no known isoforms for VEGF-C and VEGF-D, and both growth factors are identical at N- and C-termini, a feature which is not present in other VEGFs. Unlike VEGF-A and VEGF-B which are formed by alternative splicing of the primary RNA, VEGF-C and VEGF-D are produced by proteolytic processing [28,29]. These precursors are cleaved by the furin protease via a two-step process [11,23]. An initial proteolysis step produces premature variants which bind and activate both VEGFR2 and VEGFR3. However, these VEGFs have higher affinity towards VEGFR3 and very low affinity for VEGFR2. A second proteolysis step produces the mature VEGF forms that have high affinity for both VEGFR2 and VEGFR3 [30]. VEGF-C is essential for the sprouting of lymphatic vessels from the embryonic vein, thereby it is crucial in lymphangiogenesis. However, it is also involved in promoting lymphangiogenesis in various types of cancers [31]. In VEGF-C knockout mice, embryo lymphatic lineage was observed but development of lymphatic vessels were not seen, with embryonic lethality at a late stage due to a lack of lymphatic vessels [32].

Similarly, mature VEGF-D binds to both VEGFR2 and VEGFR3, therefore promoting both angiogenesis and lymphangiogenesis. Expression of VEGF-D by cancer cells is known to promote metastasis [33]. There are no known isoforms for VEGF-D, it is present in most tissues but more adequately in skin and lungs. Interestingly, out of all the VEGFs expressed in humans, VEGF-D is the only growth factor that is dispensable, as VEGF-D knockout mice were healthy, fertile with normal body mass and no abnormalities in lymphatic vessel development or function were observed [34].

### 2.4. PIGF

Placental growth factor (PIGF) is predominantly expressed in placental tissues. The human *PIGF* gene has 7 exons, and alternate RNA splicing produces four isoforms, such as PIGF-1 (PIGF_131_), PIGF-2 (PIGF_152_), PIGF-3 (PIGF_203_) and PIGF-4 (PIGF_224_) (Figure 1B). All isoforms of PIGF recognise and bind to VEGFR1, but not VEGFR2 and VEGFR3 [13,35]. Deletion of *PIGF* impairs angiogenesis, inflammation and wound healing [35,36]. However, PIGF upregulation is associated with pathological angiogenesis [37]. 

### 2.5. VEGF-E

The VEGFE gene is absent from animal species but is found in parapoxvirus, which infects sheep (*Ovis aries*) but rarely *Homo sapiens*. VEGFE encodes four splice isoforms: VEGF-E_NZ-2_, VEGF-E_NZ-7_, VEGF-E_NZ-10_ and VEGF-E_D1701_, which only bind to VEGFR2 and act as pro-angiogenic factors by promoting pathological angiogenesis in sub-cutaneous lesions infected by the virus [38]. Even though VEGF-E selectively binds to VEGFR2, the amino acid sequence of VEGF-E is less than 25% identical to VEGF-A [39]. Unlike VEGF-A, all isoforms of VEGF-E selectively bind to VEGFR2 but not VEGFR1. Thus, VEGF-E isoforms have the potential to be candidates for selectively targeting VEGFR2-specific responses towards pro-angiogenic therapy.

### 2.6. VEGF-F

VEGF-F is present in the venom of some vipers, such as *Trimereserus flavoviridis,* but not in *H. sapiens*. VEGF-F has no isoforms and selectively binds to VEGFR2, which leads to vascular permeability but very weak cell proliferation. The sequence of VEGF-F is ~50% identical to that of VEGF-A [11]. VEGF-F is however more potent than VEGF-A, with both in vivo and in vitro studies having shown that the heparin-binding domain at the C-terminus of VEGF-F competitively inhibits VEGF-A binding to VEGFR2 [40]. 

### 2.7. VEGFR1

Human VEGFR1 contains 1312 amino acids and is present as a mature ~180 kDa glycoprotein expressed in the endothelium, immune cells, epithelial and neural tissues. VEGFR1 binds all isoforms of VEGF-A, VEGF-B and PIGF. VEGF-A bind to VEGFR1 with ~2–10 picomolar (pM) affinity (*K_d_*), which is much higher than VEGFR2, while the binding affinity of PIGF for VEGFR1 is ~170 pM (*K_d_*) (Figure 1A) [41]. VEGFR1 expression is upregulated by hypoxia involving the HIF-1 complex [42]. The VEGFR1 promoter also contains a hypoxia-responsive element (HRE) sequence which enables HIF-1 binding to stimulate transcription of the VEGFR1 locus [43]. VEGFR1 knockout mice exhibit embryonic lethality and die in mid-gestation due to abnormal blood vessel formation and excessive endothelial cell proliferation [44]. Interestingly, mice carrying a mutant VEGFR1 locus lacking a functional tyrosine kinase domain show normal development and angiogenesis with no vascular defects [45]. There is an alternative soluble form (soluble VEGFR1, sFlt1) of VEGFR1, a splice variant which acts as an inhibitor for VEGFR activity [46]. The soluble splice variant acts as a decoy receptor and a negative regulator for VEGFR2 by binding to VEGF-A, thereby decreasing local concentrations of growth factor and limiting VEGF-A binding to VEGFR2 [23].

### 2.8. VEGFR2

Human VEGFR2 contain 1337 amino acids and the mature protein is a ~200–230 kDa glycoprotein expressed in both vascular and lymphatic endothelial cells. VEGFR2 binds with high affinity to VEGF-A, VEGF-C, VEGF-D and VEGF-E. Although VEGFR2 homodimers are implicated in functional regulation, VEGF-A binding can also promote VEGFR1 and VEGFR2 heterodimer formation [47,48]. A computational study showed that VEGFR heterodimers are formed at the expense of homodimers, and this also occurs when the VEGFR2 population is in excess compared to VEGFR1, with only 10–50% of VEGFR1-VEGFR2 heterodimers being functionally active [49]. The binding affinity of VEGF-A towards VEGFR2 is ~100–400 pM (*K_d_*) which is 10–100-fold lower affinity than VEGFR1 [41]. However, VEGFR1 levels are relatively low, with tyrosine kinase activity at least ten-fold lower compared to VEGFR2 in endothelial cells; therefore, it can be argued that VEGFR2 homodimers are the major signal transducers upon VEGF-A binding [50]. VEGFR2 knockout mice die mid-gestation with no organised blood vessels observed in the embryo, which are normally seen at the mid-gestation period. This is unlike VEGFR1 knockout mice, where an abnormal and irregular vascular network also causes embryonic lethality [51,52]. A soluble form of VEGFR2 (sVEGFR2) has been identified in mouse and human plasma [53]. The distinct function of sVEGFR2 is still unclear, although recent studies suggest a connection between breast cancer and sVEGFR2 levels, where higher levels of sVEGFR2 in plasma could increase the risk of breast cancer [54]. 

### 2.9. VEGFR3

Human VEGFR3 comprises 1363 residues, producing a mature ~195 kDa glycoprotein heterodimer which binds to VEGF-C and VEGF-D with high affinity. The newly translated VEGFR3 polypeptide undergoes complex processing along the secretory pathway: proteolytic cleavage generates a soluble α and membrane-bound β subunits that are linked by a disulphide bond (Figure 1A). There are two splice variants of VEGFR3 which are primarily expressed in lymphatic endothelial cells [55]. VEGFR2-VEGFR3 heterodimers have been implicated in unique signalling outcomes [56]. However, VEGFR1 and VEGFR3 heterodimer formation is unlikely [57]. Unlike VEGFR1 and VEGFR2, VEGFR3 is predominantly involved in lymphangiogenesis and not known to play a significant role in angiogenesis. VEGFR3 KO mice exhibit defective blood vessel formation in mouse embryos. Even though vasculogenesis and angiogenesis were observed in VEGFR3 KO mice, blood vessels were abnormally organised with defective lumen, which resulted in cardiovascular failure in the mouse embryo [58]. Similar to VEGFR1 and VEGFR2, VEGFR3 exists as a soluble variant [54]. sVEGFR3 is known to be expressed in corneal endothelial cells: the sVEGFR3 found in the cornea has anti-lymphangiogenic properties and binds to both VEGF-C and VEGF-D, potentially acting as a decoy receptor for both ligands. This could block the lymphangiogenesis promoted by VEGFR3 signal transduction [59]. sVEGFR3 is also involved in supressing allo-sensitization and promoting survival of corneal allografts [60].

## 3. Structural Features of VEGFRs and Their Functions

All VEGFRs display a similar primary structure with seven immunoglobulin (Ig)-like extracellular immunoglobulin domains, a short α-helical transmembrane domain, followed by a cytoplasmic juxtamembrane region, then a tyrosine kinase (TK) domain linked to a flexible carboxyl-terminus or tail [61] (Table 1). A generally accepted view is that VEGFR binding to cognate ligands trigger the formation of homodimers, activating cell signalling pathways that leads to various cellular functions. 

### 3.1. VEGFR Extracellular Domain

Non-activated or resting VEGFR molecules are thought to be monomeric polypeptides. However, mathematical modelling [50] and binding studies [62] postulate the presence of non-activated VEGFR monomers and dimers. Similar to other RTKs, VEGF binding to the VEGFR extracellular domain triggers dimer formation, activation and downstream signalling [7]. Dimer formation is associated with trans-autophosphorylation of specific tyrosine residues within the cytoplasmic domain, including the juxtamembrane, tyrosine kinase and tail regions [63,64]. The extracellular domain consists of seven Ig-like extracellular immunoglobulin domains, which are mainly involved in ligand binding and receptor dimerization, and such interactions have been extensively studied using structural approaches. Crystal structures of soluble Ig-like domains derived from all three VEGFRs with or without bound VEGF have been solved (Table 2). Ballmer-Hofer and colleagues recently presented a high-resolution analysis of the structure of a soluble VEGF-A/VEGFR1 complex revealing ligand-induced dimerization of the VEGFR1 extracellular domain with homotypic interactions between Ig-like domains 4, 5 and 7 [65]. In both VEGFR1 and VEGFR2, extracellular domains 2 (D2) and 3 (D3) are critical for VEGF-A recognition: ligand binding triggers conformational changes that are postulated to be transmitted through the transmembrane region to cause TK activation [66]. For VEGFR2, the interface between VEGF-A and D2 is hydrophobic, whereas VEGF-A binding to D3 involves hydrophilic interactions [67]. VEGFR3 binding to ligands is similar but the binding sites extend over D1 to D3; furthermore, D4–D7 contribute to structural rearrangements crucial for VEGFR3 dimerization and activation [68]. Rahimi and colleagues have shown that an RTK chimera comprising the colony-stimulating factor-1 receptor (CSF-1R) extracellular domain fused to the VEGFR2 transmembrane and cytoplasmic domain can be activated by CSF-1 with similar signalling outcomes [69]. Thus, VEGF-stimulated TK activation via ligand binding to the VEGFR extracellular domain is part of a widely conserved mechanism across the RTK family.

### 3.2. VEGFR Transmembrane Domain

The α-helical transmembrane domain (TMD) in VEGFRs undergo non-covalent oligomerisation within the lipid bilayer, which also influences RTK complex formation and stability. Interestingly, VEGFR activation is dependent on the orientation of transmembrane α helices. Replacement of the native VEGFR2 TMD with a synthetic TMD carrying glutamic acid residues promotes TMD dimer formation, suggesting that TK activation is dependent on orientation and oligomerisation of these regions within VEGFR2. Further work by Dosch and colleagues showed that VEGFR2 TMD mutants containing glutamic acid residues spaced at 7 amino acids apart showed ligand-independent activation and dimerization [80]. Such studies have shown that a ligand is not necessary for activation of VEGFRs; however, they support the view that VEGF ligand binding causes a change in VEGFR monomer orientation within a dimeric complex, thus influencing substantial rearrangement of the cytoplasmic domains relative to each other. 

### 3.3. VEGFR Cytoplasmic Domain

Each VEGFR cytoplasmic domain has a juxtamembrane domain, a ‘split’ tyrosine kinase domain, followed by flexible carboxyl-terminus or ‘tail’. The VEGFR region located immediately after the TMD is termed the juxtamembrane domain (JMD). The JMD has been shown to play a role in activation and repression of TK activity, as replacement of the VEGFR1 JMD with equivalent VEGFR2 JMD caused a significant increase in VEGFR1 TK activity [81]. In response to VEGF-A stimulation, this mutant VEGFR1 now caused increased PI3K activation and endothelial cell migration [81].

**Figure 2:**
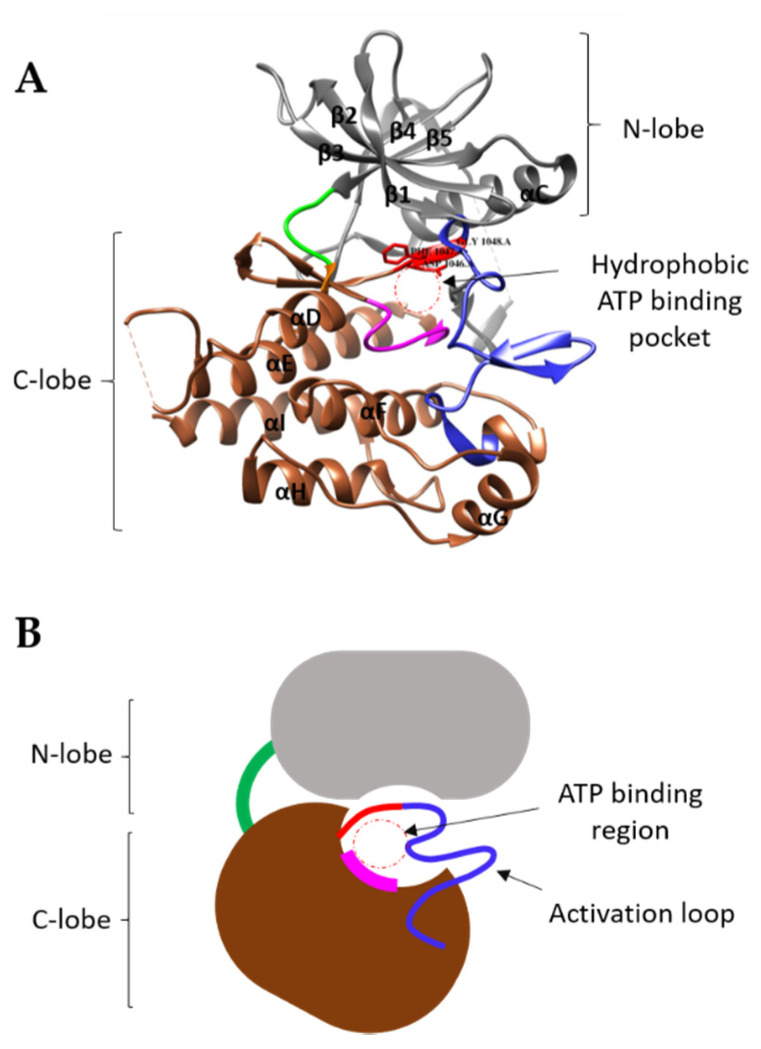
(**A**) Crystal structure of VEGFR2 kinase, N-lobe is shown in grey, C-lobe in brown, the hinge region which connects the N- and C-lobes is shown in green, catalytic loop in magenta and the activation loop in blue, with conserved DFG (D1046–F1047–G1048) motif of activation segment in red. Both α-helices and β-sheets are labelled from αC–αI and β1–β5, respectively. (PDB code: 4AGD). (**B**) Schematic representation of inactive VEGFR kinase showing the adenosine triphosphate (ATP) binding region (red circle), catalytic loop (magenta), DFG motif (red) and closed activation loop (blue). The activation loop controls the access to the ATP-binding site.

The VEGFR tyrosine kinase (TK) domain of ~300 residues display an atypical ‘split’ configuration into N- and C-terminal lobes caused by the insertion of a ~70 residue sequence within the middle of this catalytic domain. An engineered VEGFR2 ‘core’ TK module lacking the insert sequence has been crystallised and a high-resolution structure complexed to different tyrosine kinase inhibitors (TKIs) has been documented (Table 2). The initial structure of the VEGFR2 TK ‘core’ shows commonality in the mode of adenosine triphosphate (ATP) binding compared to other RTKs [82]. The ATP-binding site is located between the juncture of the two N- and C-lobes. The N-lobe mainly consists of an alpha helix (αC) and five anti-parallel beta strands (β1–β5). The glycine-rich region (841–846) is present in the N-lobe and has a hydrophobic aromatic residue (F845) which is positioned close to the ATP-binding site (Figure 2A). The C-lobe is much larger in comparison to the N-lobe and consists of seven α-alpha helices (αD-αI and αEF), four short β-strands (β6–β9), a catalytic loop and an activation region. The catalytic loop contains a conserved HRD (H1026–R1027–D1028) motif, whereas the activation segment starts with the conserved DFG motif (D1046–F1047–G1048) and ends with APE (A1073–P1074–E1075). For a large majority of protein kinases, the DFG and APE motifs regulate kinase activity. VEGFR2 also displays the KEDD (K868–E885–D1028–D1046) motif, which modulates TK activity and TKI binding [83]. 

## 4. Mechanism of TK Activation 

**Figure 3:**
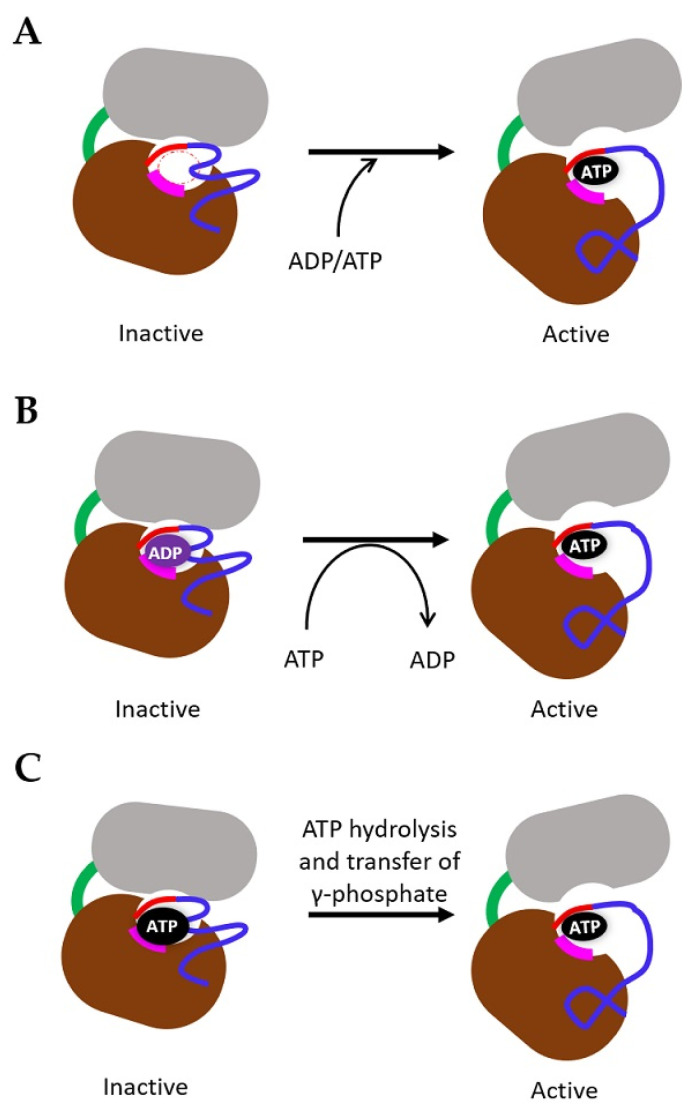
Hypothetical schematic models of VEGFR kinase activation. (**A**) Inactive kinase domain with vacant adenine nucleotide binding site, activation of receptor stimulates the change in orientation of N- and C-lobes by allosteric regulation between the hinge region and the activation loop which give access for adenosine diphosphate/adenosine triphosphate (ADP/ATP) to the binding site, thereby activating the kinase. (**B**) Inactive kinase domain bound to ADP, conformational change in kinase domain leads to exchange of ADP with ATP, similar to the guanosine diphosphate/guanosine triphosphate (GDP/GTP) exchange by Ras protein. This may potentially lead to the activation of kinase. (**C**) Inactive kinase domain with the adenine binding site containing the ATP molecule, stimulation of receptor potentially leads to hydrolysis of ATP and releases the γ-phosphate to phosphorylate the tyrosine residues, thereby activating the kinase domain.

Many studies performed using different approaches have tried to understand how ligand binding to RTK causes conformational changes which regulate TK activation. Using both generic models [84], EGFR [85,86] and VEGFRs [30,64,65] structural models, we consider the specific role of ADP/ATP in directing resting/inactive or active TK states. We sketch 3 hypothetical models in this context (Figure 3). In Model 1, the TK domain is ‘empty’ of either ADP or ATP and requires a directed stimulus to load the adenine nucleotide-binding site with either ADP or ATP. In Model 2, the TK domain is in an ADP-bound inactive state and requires some form of stimulus and conformational change to exchange ADP for ATP. This scenario is similar to that for small G-proteins such as Ras, where cycling between GDP/GTP-bound forms regulates inactive/active signalling [87,88]. In Model 3, due to the 100–200-fold excess in ATP cytosolic concentration over ADP, the TK domain is in an ATP-bound state, but is held in an inactive state and requires a stimulus to hydrolyse ATP and transfer the g-phosphate onto a target site or substrate. The EGFR-L834R oncogenic mutant appears to increase TK activity and enhance dimer formation [89]. Moreover, one reaction of these patients to EGFR inhibitor cancer therapy is the T766M mutation that increases the affinity of the EGFR-L834R oncoprotein by more than 10-fold [90].

Within the VEGFR family, the intervening activation loop that separates the N- and C-lobes is lengthened by the addition of the 70-residues insert common to all 3 membrane proteins. Sequence conservation in this kinase domain insert is low; however, there are additional tyrosine residues within each insert sequence which could enable different modes of TK activation. Spacing of these tyrosine residues within the insert sequence is not conserved between the three VEGFRs, suggesting that the presence of phosphotyrosines in this region could have different consequences for TK activity. VEGFR2 undergoes phosphorylation at the unique Y951 residue located within the insert region, which stimulates recruitment of adaptor protein TSAd and downstream activation of c-Src tyrosine kinase [91]. 

**Figure 4:**
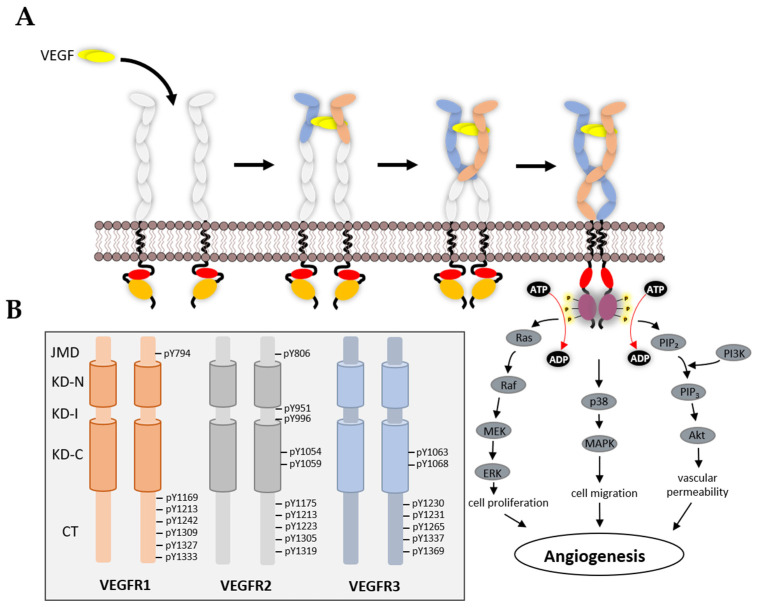
(**A**) Schematic representation of mechanism of VEGFR activation and downstream signalling pathways. A covalently linked VEGF dimer (yellow) binds to second and third extracellular immunoglobulin (IgG)-like domains of VEGFR that leads to dimerization of receptors with homotypic contacts between the fifth and seventh IgG-like domains. The complex is also stabilised with additional contacts at transmembrane and juxtamembrane regions that leads to phosphorylation of various tyrosine kinase residues that trigger downstream pathways such as the Ras-Raf-MEK-Erk pathway, p38-MAPK pathway and PI3K-Akt pathway. (**B**) Location of various tyrosine residues that undergo phosphorylation in three different VEGFRs. Domains are juxtamembrane domain, JMD; kinase domain-N-lobe, KD-N; kinase domain insert, KD-I; kinase domain-C-lobe, KD-C; and C-terminal tail, CT.

All individual Ig-like domains’ structures indicate that a VEGF homodimer binds to two VEGFR chains with a 1:1 stoichiometry. The structure of a soluble truncated VEGFR1 chain bound to VEGF-A indicates that homotypic interactions between VEGFR1 domains D4, D5 and D7 are likely to also influence transmission of conformational changes towards the VEGFR cytoplasmic domain, thus influencing TK activation (Figure 4A) [65]. Ballmer-Hofer and colleagues have used synthetic protein technology to generate ankyrin repeat proteins (DARPins) that bind to VEGFR2 extracellular domains [92]. DARPins specific for VEGFR2 domains D4 and D7 can block the TK activation and downstream signalling but did not prevent VEGF ligand binding nor VEGFR2 homodimer formation [92]. Another synthetic protein called Affimer has been used to target the VEGFR2 extracellular domain to block VEGF-A-stimulated signal transduction, cell migration and tubulogenesis. 

Such VEGF ligand-induced VEGFR dimerization linked to torsional changes in the extracellular domain is likely to bring the TK domain into an active conformation through allosteric regulation, which could allow both N- and C-lobes to ‘open’ or flex to either allow ATP access, or stimulate ADP exchange for ATP. It is unclear whether an inactive, resting or monomeric RTK has an empty site, or whether this is always occupied by either ADP or ATP. As the cytosolic concentration of ATP (3–5 mM) is approximately 100–200-fold greater than ADP levels, ATP/ADP exchange for each RTK is likely dependent on tight regulation of TK conformation, and allowing ATP accessibility to the binding site within the molecule. An alternative scenario is that a monomeric or dimeric RTK complex such as VEGFR is largely ATP-bound (driven by the concentration gradient), but the activation of the TK activity is somehow restricted until the VEGF ligand binds.

Both N-terminal and C-terminal lobes are connected by the flexible insert which acts as a ‘hinge’ with the hydrophobic ATP-binding pocket formed by residues from both the N- and C-lobes [93,94]. There are 5–7 tyrosine phosphorylation sites within each VEGFR which are typically distributed within the JMD, insert and tail regions, however, some sites do also occur within the N- and C-lobes. Within the cleft and the ATP-binding site of the VEGFR2 TK ‘core’, the adenine ring of the ATP molecule forms hydrogen bonds with the hinge region, whereas the ribose sugar and triphosphate moieties are coordinated via the conserved DFG and APE motifs essential for regulating TK activity [95]. In protein kinases, the connecting activation loop (VEGFR2 residues 1046–1075) also plays a crucial role in unblocking the substrate binding site within the cleft, thereby enabling transfer of the g-phosphate from ATP onto the –OH group of the tyrosine side chain [95]. The expansion of this loop region by ~70 residues within the VEGFR kinase domain suggests a more flexible juxtaposition of N- and C-lobes. Furthermore, binding of adaptor proteins to the phosphorylated VEGFR2-Y951 epitope indicates that such interactions could act to stabilise this loop region and may indirectly modulate TK activity [91]. 

A central feature of protein kinase regulation is the juxtaposition of a C-terminal tail to modulate kinase activity [84]. The looping of a flexible tail sequence across the kinase domain could potentially act to stabilise an inactive or active state. A conserved phosphotyrosine epitope within the tail region in VEGFR1 (Y1169) and VEGFR2 (Y1175) enables recruitment of PLCγ1 to the activated VEGFR. Furthermore, other phosphorylation sites in the VEGFR1 (Y1213, Y1333) and VEGFR2 (Y1214) may also function to recruit other effectors. Studies suggest that VEGFR2-mediated recognition of soluble or extracellular matrix-bound VEGF-A elicits differential phosphorylation of Y1214 and association with β1 integrin. Furthermore, the phosphorylation of the VEGFR2-Y1214 epitope is linked to downstream activation of the p38 mitogen-activated protein kinase (MAPK) pathway [96]. In contrast, different adaptors are postulated to be recruited to activate VEGFR1 upon phosphorylation of residue Y1213 [97]. 

The flexible carboxyl tail (~200 residues) is the least conserved region within the VEGFR cytoplasmic domain and may mediate both positive and negative regulation of tyrosine kinase activity. When the VEGFR1 carboxyl terminal tail is swapped with the VEGFR2 tail, this causes an increase in ligand-dependent auto-phosphorylation, VEGFR1 downregulation and endothelial cell proliferation [98]. Furthermore, deletion of the carboxyl-tail of VEGFR2 eliminated its ability to activate signalling proteins and cell proliferation, therefore inhibiting VEGFR2 tyrosine kinase activity. However, when the VEGFR2 tail is replaced with the VEGFR1 tail, this VEGFR chimera showed restoration of ligand-stimulated signalling and cell proliferation [99]. This indicates that the carboxyl-terminal tail of VEGFR1 acts as a negative regulator of kinase activity, while the VEGFR2 tail acts as a positive regulator.

By comparison to EGFR, it is postulated that, whether this RTK is in monomeric or dimeric states, the C-terminal tail is relatively free [86]. However, different studies suggest that inactive EGFR dimers and tetramers exist [100], and these may require tight packing of the C-terminal tail within these complexes. One view is that EGFR dimers are inactive and the transition to higher order EGFR tetramers is required for an active signalling complex which mediates recruitment of adaptors such as Grb2 [101,102]. It also raises the question of how symmetric or asymmetric arrangement of the kinase domains within a dimer influences the activated TK state.

## 5. VEGFR Signal Transduction

The activated VEGFR recruits and phosphorylates adaptors, enzymes and effectors that affect a wide variety of cellular responses in different cells and tissues. Although VEGFR1 is largely postulated to be a negative regulator of VEGF-A-regulated signal transduction, it can undergo phosphorylation at specific tyrosine residues (Y794, Y1169, Y1213, Y1242, Y1309, Y1333) (Figure 4B), which enables it to interact with adaptor proteins and phospholipase C gamma-1 (PLCγ1) [103,104,105]. PLCγ1 recruitment and phosphatidylinositol 4,5-bisphosphate (PIP_2_) hydrolysis leads to the production of IP_3_ (calcium flux) and diacylglycerol (PKC activation), which has a wide variety of effects on cellular pathways and responses. There is evidence that VEGFR1 activation regulates cell migration and vascular permeability, and one likely downstream target is the PI3K/Akt pathway [106]. PIGF binding stimulates VEGFR1-Y1309 phosphorylation, leading to downstream Akt activation [35], suggesting that different VEGF ligands cause different patterns of VEGFR activation, protein recruitment and downstream signalling.

VEGFR2 is the major RTK that enables VEGF-regulated angiogenesis. The activation of VEGFR2 leads to multiple phosphorylation sites (Y801, Y951, Y996, Y1054, Y1059, Y1175, Y1214, Y1319) (Figure 4B). Phosphorylation of Y801 in the JMD is implicated as an early step in stimulating maximal TK activity of VEGFR2 [107]. Furthermore, phosphorylation of VEGFR2-Y801 is linked to PI3K/Akt signalling and downstream activation of endothelial nitric oxide synthase (eNOS) [108]. In endothelial cells, appearance of the VEGFR2-pY1059 is linked to cytosolic Ca^2+^ flux, MAPK activation and cell proliferation, whereas the VEGFR2-pY951 epitope is linked to increased cell migration [109]. The VEGFR2-pY1175 epitope is a key signature as a result of VEGF-A binding and creates a binding site for PLCγ1 [110]. Recruitment of PLCγ1 to the plasma membrane leads to PIP_2_ hydrolysis, and one consequence is activation of the canonical MAPK pathway linked to cell proliferation [110,111]. 

VEGFR3 is responsible for lymphangiogenesis. Activation of VEGFR3 leads to phosphorylation of residues Y1063, 1068, 1230, 1231, 1265, 1337 and 1363 (Figure 4B) [64]. Residues Y1063 and Y1068 are located within the TK catalytic region, whereas the remaining phospho-epitopes are within the flexible tail region. VEGFR3 activation and tyrosine phosphorylation can activate PI3K/Akt, PKC and MAPK signal transduction pathways [20]. 

## 6. Strategies Employed in Inhibition of VEGFR Function

The phenomenon of angiogenesis is not only a physiological process but can also contribute to pathological conditions such as tumour growth and progression. Targeting pro-angiogenic output by VEGFRs is thus important in cancer therapy. Different molecules can act as anti-angiogenic drugs that block VEGFR signal transduction and have also been clinically approved for cancer and wet age-related macular degeneration (AMD). Here, we discuss the molecular basis of current strategies for translating VEGFR inhibition towards clinical care. 

### 6.1. Protein-Based Therapies

Humanised monoclonal antibodies that bind to the circulating VEGF-A with high affinity can prevent interaction with VEGFRs, block endothelial responses and tumour neovascularisation (Table 3) [112]. Bevacizumab (Avastin) is a humanised IgG_1_ monoclonal antibody that binds all VEGF-A isoforms with 58 pM (*K_d_*) affinity to VEGF-A _165_ and blocks VEGFR2 signalling [113,114]. Bevacizumab is clinically approved as part of multimodal treatments for advanced non-small cell lung cancer (NSCLC), advanced colorectal cancer (CRC), metastatic breast cancer, renal cell cancer and advanced glioblastoma multiforme [115,116]. Ranibizumab is a humanised antibody based on a single antigen-binding site (Fab) derived from Bevacizumab but has much higher VEGF-A binding affinity (46 pM (*K_d_*)) [113]. The original Bevacizumab has divalent binding sites for VEGF-A but Ranibizumab is a monovalent species [114,117]. Ranibizumab (Lucentis) is clinically approved for use in ocular diseases involving aberrant angiogenesis such as wet AMD: the smaller monovalent Fab molecules more easily diffuse into the ocular environment compared to full-size antibodies [117,118].

Another approach is to directly target the VEGFR extracellular domain, thus modulating interaction with VEGF ligand and signalling outcomes. Ramucirumab is a humanised IgG_1_ monoclonal antibody that binds to the VEGFR2 and is approved for advanced gastric cancer [119] and NSCLC [120]. Ramucirumab binds at or close to the VEGF-A binding site on VEGFR2 and blocks ligand binding and VEGFR2 activation [126,127]. Several anti-VEGFR2 humanised antibodies are in pre-clinical development and clinical trials. Similar to Ramucirumab’s mode of action, Tanibirumab (TTAC-0001) is an anti-VEGFR2 antibody which also binds to the VEGFR2 extracellular domain and blocks binding of VEGF-A, VEGF-C, VEGF-D and VEGF-E, with potent anti-angiogenic activity and tumour growth inhibition in mouse models. Tanibirumab shows effective inhibition of VEGFR2 but not VEGFR1 or VEGFR3 [128]. Tanibirumab shows positive results in patients with colorectal cancer in phase I clinical trials, and phase II trials are ongoing [129]. 

The use of synthetic proteins is being increasingly explored to target angiogenesis in disease states. One such example is Aflibercept (Zaltrap, VEGF TrapEye), which contains the high-affinity VEGF-A binding site from VEGFR1, fused to dimerization domains from VEGFR2, with a humanized Fc portion to recruit the immune system [113,121]. Such a construct in effect acts as a ‘VEGF ligand trap’ that inhibits angiogenesis. Aflibercept also binds to other VEGF family members, including VEGF-A (*K_d_* ~ 0.49 pM), VEGF-B and PIGF, and reduces activation of both VEGFR1 and VEGFR2. The functional effects of Aflibercept in decreasing vascular permeability and neovascularisation [121,122] have led to clinical approval to treat wet AMD [123]. 

Anti-VEGFR1 therapy has also shown promise with Icrucumab (IMC-18F1), being able to inhibit human breast tumour xenograft growth in mice [130]. Multimodal treatment combining Icrucumab with a nucleotide analogue (Capecitabine) is being used in phase II trials on patients with locally advanced or metastatic breast cancer [131]. Another monoclonal antibody (D7F16) against VEGFR1 [132] has been shown to inhibit ligand-dependent VEGFR1 homodimerization and activation in human glioblastoma and glioblastoma stem cells [133]. A VEGFR3-specific monoclonal antibody (HF4-3C5) acts as an antagonist by inhibiting VEGF-C binding [134]. Furthermore, a bispecific chimera antibody (diabody) combining VEGFR2-specific (IMC-1121) and VEGFR3-specific (HF4-3C5) binding properties has been developed. This unique diabody blocks VEGFR2 and VEGFR3 activation simultaneously [135].

The use of a synthetic polynucleotide or protein polymers is also promising. Pegaptanib is a pegylated nucleic acid polymer that binds with high affinity to VEGF-A isoforms implicated in tumour neovascularisation and vascular permeability, but does not recognise other VEGF-related family members [124]. Pegaptanib has been clinically approved for treating wet AMD, a leading cause of age-related blindness [125]. Interestingly, different approaches have identified VEGFR2-specific agents based on synthetic protein scaffolds called DARPins [92] and Affimers [136]. In both studies, targeting the VEGFR2 extracellular domain can block ligand-regulated signal transduction and endothelial cell responses [92,136]. Such studies provide hope for new, more effective cancer therapies based on selective targeting of ligand or receptor domains that inhibit disease-related outcomes but do not affect normal physiological function.

### 6.2. Tyrosine Kinase Inhibitors (TKIs)

**Figure 5:**
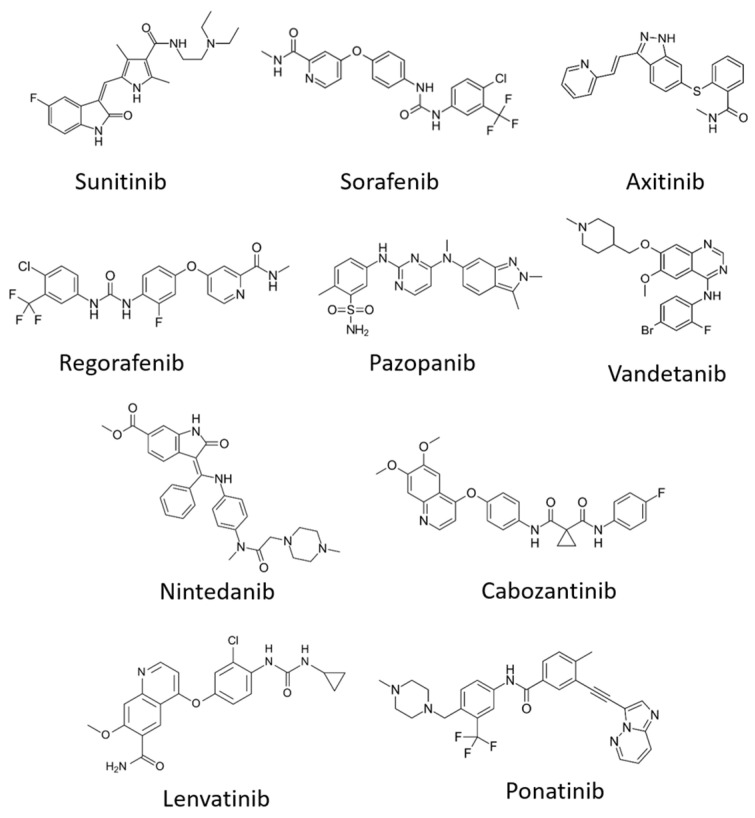
Chemical structures of U.S Food Drug and Administration (FDA)-approved small-molecule tyrosine kinase inhibitors that target VEGFRs.

Small molecules called tyrosine kinase inhibitors (TKIs), which are water-soluble and have amphipathic properties, enable passage through the plasma membrane bilayer to reach their target site within the cell. By binding to the RTK tyrosine kinase domain, such molecules can perturb TK activity, which is dependent on the ATP/ADP cycle (Figure 5, Table 4). The ATP molecule comprises of the adenine ring, a ribose sugar and three phosphate groups. Within the TK catalytic domain, the adenine-binding region is located between hydrophobic pockets 1 and 2, and hydrogen bonds stabilise contacts between the adenine ring and the hinge region [137]. The hydrophilic ribose moiety and the negatively charged phosphates bind to conserved residues essential for catalysis. TKIs generally act by competitive inhibition with ATP for binding to the TK domain. Most TKIs mimic the adenine moiety by forming hydrogen bonds within the TK hinge region but generally lack the ribose or phosphate binding properties [95].

We shall focus on VEGFR-specific TKIs, which can be classified differently based on their mechanism of action. Type I molecules only recogniae the active TK conformation: they are ATP mimetics which form between 1 to 3 hydrogen bonds within the adenine-binding site, thereby blocking ATP binding in a competitive manner. Type I TKIs are generally non-selective and inhibit a broad range of kinases due to the highly conserved mechanism of action. The majority of ATP competitive inhibitors are type I TKIs. Sunitinib (Sutent) is one such example and is a VEGFR TKI that has been clinically approved for cancer therapy (Figure 5). In contrast, type II TKIs recognise the inactive TK conformation. The ‘DFG out’ conformation is an inactive state created by the activation loop, with an exposed hydrophobic pocket adjacent to the ATP-binding site. Type II TKIs indirectly compete with ATP by filling this exposed hydrophobic pocket, thereby inhibiting ATP binding by steric hindrance. In contrast to non-selective type I TKI action, type II TKI displays more selectivity, as the ‘DFG out’ inactive conformation exhibits variability between TKs [152]. The clinically approved drugs, Imatinib and Sorafenib, are examples of type II TKIs. Compounds with covalent modification ability contribute to class III TKIs, with the modification of specific cysteine residues within the catalytic TK domain causing irreversible changes in RTK function. The class III TKI usually has an electrophilic group which forms a covalent bond with the electron-rich sulphur moiety within the cysteine side chain. This irreversible conjugation of the cysteine side chain within the TK domain can block ATP binding, TK activation and is usually highly selective. Vandetanib (ZD6474) targets VEGFR2 strongly and VEGFR3 more weakly [95]. This compound can also modify other RTKs such as EGFR and RET, again indicating that many such drugs lack single target selectivity.

**Figure 6:**
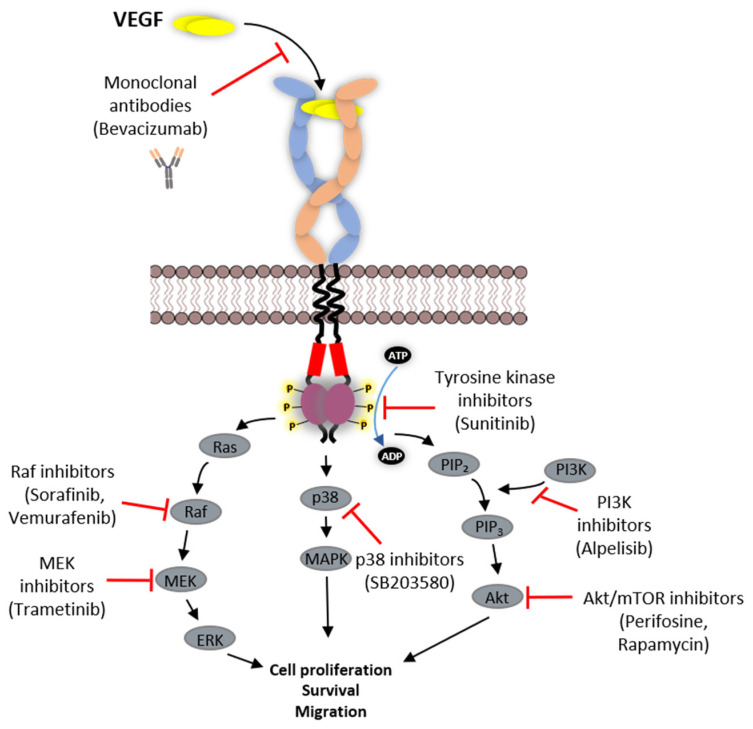
Schematic representation of strategies employed in targeting VEGFRs. VEGFRs are targeted directly by monoclonal antibodies to inhibit VEGF binding to the extracellular domain of the receptor or using various small-molecule tyrosine kinase inhibitors which block the ATP binding in the kinase domain, thereby blocking phosphorylation of tyrosine residues. Alternatively, various small-molecule inhibitors were developed that target cell signalling pathways activated by VEGFRs.

One hope is that new developments in class IV protein kinase inhibitors will allow more selective targeting of RTK activity and function. Class IV inhibitors behave in an allosteric manner to negatively regulate protein kinase by distinctly binding outside the ATP-binding site. By such a mode of action, it should be theoretically possible to obtain selective inhibitors to any protein kinase. Such compounds have been demonstrated to target canonical MAPK, p38-MAPK pathway and mammalian target of rapamycin (mTOR) signal transduction pathways (Figure 6). Vemurafenib (PLX4032) is a B-Raf selective inhibitor that also inhibits both MEK1/2 and ERK1/2. This drug received US Food and Drug Administration (FDA) approval for treating unresectable or metastatic melanoma in patients carrying the B-Raf-V600E mutation [153,154,155]. SB203580 is a pyridinyl imidazole inhibitor that targets p38 MAPK [156] and PDK1, affecting downstream Akt activation and retinoblastoma hyperphosphorylation [157]. Perifosine is an anti-cancer molecule that inhibits the Akt pathway [158]. Dactolosib (BEZ235), Bimiralisib (PQR309), BGT226, SF1126 and GSK2126458 are some examples of class IV inhibitors developed to target the PI3K-Akt-mTOR pathway with dual inhibitory activity towards PI3K and mTOR [159]. Everolimus is an mTOR inhibitor which has received FDA approval for treating advanced or metastatic renal cell carcinoma in combination with Lenvatinib, a multi-kinase RTK inhibitor for the VEGFR subfamily [160,161,162]. Alpelisib, which inhibits PI3K, has been approved for multimodal therapy in some types of breast cancer [163].

## 7. Conclusions and Future Directions

The VEGFR-VEGF system plays central roles in embryonic and physiological angiogenesis, as well as tumour angiogenesis. Understanding how the structure, activation and functional outputs associate with each VEGFR is important for treating a wide range of human pathologies associated with this RTK subfamily. Advances in solving the structure of individual domains of VEGFs, VEGFRs and VEGFR-VEGF complexes have provided invaluable information for designing many drugs in clinical use. Targeting angiogenesis as an anti-cancer strategy has become increasingly important with the realisation that tumour growth and metastasis can be simultaneously blocked using anti-angiogenic therapies. Significant progress has been made by the generation of small-molecule TKIs, humanised antibodies, synthetic proteins and aptamers that have provided disease remission in some pathological states. 

However, drug resistance continues to be a major obstacle in anti-cancer therapy. Many VEGFR therapies are multimodal or combination therapies including some type of chemotherapy: one consequence of chemotherapy is high incidence of genetic mutations that give rise to new drug-resistant phenotypes. Tumours can develop resistance to anti-angiogenic therapy indirectly whilst the VEGFR signalling remains inhibited by VEGFR inhibitors [164]. In such cases, combination therapy with multiple TKIs could overcome drug resistance. For instance, resistance to Bevacizumab treatment in colorectal cancer (CRC) due to tolerance of hypoxia can be overcome by combination with Nintedanib, a multi-kinase inhibitor that targets VEGFRs, FGFRs and PDGFRs [165]. There is a need to understand the full structural ensemble of ligand, RTK and RTK-ligand complexes in active and inactive states to design better drugs that ‘switch off’ harmful signals into the cellular interior, whilst maintaining normal or physiological outputs. Otherwise, side effects and drug resistance will continue to be major issues that hamper the development of more effective therapeutic compounds for many ailments where the VEGFR-VEGF axis plays key roles.

## Figures and Tables

**Table 1:** Classification of different domains of vascular endothelial growth factor (VEGF) receptors.

Details	VEGFR1	VEGFR2	VEGFR3
Swiss UniProt ID	P17948	P35968	P35916
Full length	1338	1356	1363
Signal peptide	26 (1–26)	19 (1–19)	24 (1–24)
Receptor chain	1312 (27–1338)	1337 (20–1356)	1339 (25–1363)
Extracellular domain	732 (27–758)	745 (20–764)	751 (25–775)
Immunoglobulin (Ig)-like 1	92 (32–123)	65 (46–110)	98 (30–127)
Immunoglobulin (Ig)-like 2	64 (151–214)	67 (141–207)	63 (151–213)
Immunoglobulin (Ig)-like 3	98 (230–327)	97 (224–320)	108 (219–326)
Immunoglobulin (Ig)-like 4	87 (335–421)	87 (328–414)	85 (331–415)
Immunoglobulin (Ig)-like 5	126 (428–553)	128 (421–548)	131 (422–552)
Immunoglobulin (Ig)-like 6	99 (556–654)	110 (551–660)	117 (555–671)
Immunoglobulin (Ig)-like 7	87 (661–747)	87 (667–753)	87 (678–764)
Transmembrane domain	22 (759–780)	21 (765–785)	21 (776–796)
Cytoplasmic domain	558 (781–1338)	571 (786–1356)	567 (797–1363)

**Table 2:** Structural studies on VEGFR complexes and/or inhibitors.

Complex	Protein Data Bank (PDB) Code	Domains	Method	Reference
VEGFR1/VEGF-A	1FLT	Domain 2	X-ray diffraction	[70]
VEGFR1/VEGF-A	1QTY	Domain 2	X-ray diffraction	[71]
VEGFR1/PIGF	1RV6	Domain 2	X-ray diffraction	[67]
VEGFR1/VEGF-B	2XAC	Domain 2	X-ray diffraction	[72]
VEGFR1/VEGF-A	5T89	Domains 1–6	X-ray diffraction and negative stain EM	[65]
VEGFR2	3KVQ	Domain 7	X-ray diffraction	[73]
VEGFR2/VEGF-A	3V2A	Domains 2 and 3	X-ray diffraction	[74]
VEGFR2/VEGF-C	2X1W	Domains 2 and 3	X-ray diffraction	[75]
VEGFR2/VEGF-C	2X1X	Domains 2 and 3 in tetragonal crystal form	X-ray diffraction	[75]
VEGFR2 kinase domain	1VR2	Kinase domain	X-ray diffraction	[76]
VEGFR2 kinase domain with sunitinib	4AGD	Juxta membrane and kinase domains	X-ray diffraction	[77]
VEGFR2 kinase domain with sorafenib	4ASD	Juxta membrane and kinase domains	X-ray diffraction	[77]
VEGFR2 kinase domain with axitinib	4AGC	Juxta membrane and Kinase domains	X-ray diffraction	[77]
VEGFR2 kinase domain with tivozanib	4ASE	Juxta membrane and kinase domains	X-ray diffraction	[77]
VEGFR2/VEGF-E	3V6B	Domains 2 and 3	X-ray diffraction	[74]
VEGFR2/DARPin Db4	5OYJ	Domains 4 and 5	X-ray diffraction	[78]
VEGFR2 trimeric mutant transmembrane domain	2MET	Transmembrane domain	NMR	[79]
VEGFR2 mutant dimeric transmembrane domain	2MEU	Transmembrane domain	NMR	[79]
VEGFR2 dimeric membrane domain in DPC micelles	2M59	Transmembrane domain	NMR	[79]
VEGFR3/VEGF-C	4BSK	Domains 1 and 2	X-ray diffraction	[68]
VEGFR3 ECD	4BSJ	Domains 4 and 5	X-ray diffraction	[68]

**Table 3:** Clinically approved VEGF inhibitors.

Name	Target	Type	Treatment	Reference
Bevacizumab (Avastin)	VEGF-A	Human monoclonal IgG1 antibody	Non-small cell lung cancer (NSCLC), colorectal cancer, metastatic breast cancer, renal cell cancer and advanced glioblastoma multiforme	[115,116]
Ranibizumab (Lucentis)	VEGF-A	Antibody Fab fragment	Corneal neovascularisation	[117,118]
Ramucirumab (Cyramza)	VEGFR2	Human monoclonal IgG1 antibody	Advanced gastric cancer and non-small cell lung cancer (NSCLC)	[119,120]
Aflibercept (Zaltrap)	VEGF-A, VEGF-B and PIGF	Soluble decoy receptor	Neovascular age-related macular degeneration (AMD), Diabetic macular edema (DME)	[121,122,123]
Pegaptanib (Macugen)	VEGF 165	Polynucleotide aptamer	Neovascular age-related macular degeneration (AMD)	[124,125]

**Table 4:** Clinically approved small-molecule VEGFR inhibitors.

Inhibitors	VEGFR1	VEGFR2	VEGFR3	Treatment	Other Targets	References
Sunitinib (Sutent)	+	+	+	Metastatic renal cell carcinoma (mRCC) and gastrointestinal stromal tumour (GIST)	PDGFRβ, FLT3	[138,139]
Sorafenib (Nexavar)		+		Advanced renal cell carcinoma, advanced hepatocellular carcinoma	PDGFR, c-kit, Raf-1, B-Raf	[140,141]
Cabozantinib (Cabometyx)		+		Advanced renal cell carcinoma	MET, RET	[142,143]
Pazopanib (GW78603)	+	+	+	Renal cell carcinoma	PDGFR-α, PDGFR-β and c-kit	[144]
Ponatinib (AP24534)		+		Chronic myeloid leukaemia	FGFR-1, FGFR-2, FGFR-3, PDGFR-α	[145]
Regorafenib (BAY 73-4506)	+	+	+	Metastatic colorectal cancer (mCRC), gastrointestinal stromal tumours (GIST) and hepatocellular carcinoma	FGFR-1, FGFR-2, PDGFR-α, PDGFR-β, KIT, TIE2, TrkA	[146,147]
Axitinib	+	+	+	Renal cell carcinoma	PDGFRβ, c-Kit	[148]
Lenvatinib (E7080)	+	+	+	Radioactive iodine (RAI)-refractory thyroid cancer	PDGFR-α, FGFR-1, FGFR-2, FGFR-3, FGFR-4, KIT and RET	[149,150]
Vandetanib (ZD6474)		+	+	Locally advanced and metastatic medullary thyroid cancer	EGFR, RET	[151]
Nintedanib (BIBF 1120)	+	+	+	Idiopathic pulmonary fibrosis (IPF)	FGFR-1, FGFR2, FGFR3, PDGFR-α, PDGFR-β and FLT3	[138]
